# Booster effect of a third mRNA‐based COVID‐19 vaccine dose in patients with myeloid malignancies

**DOI:** 10.1002/cam4.6314

**Published:** 2023-07-06

**Authors:** Akio Mori, Masahiro Onozawa, Mirei Kobayashi, Shihori Tsukamoto, Hajime Senjo, Takashi Ishio, Emi Yokoyama, Minoru Kanaya, Koh Izumiyama, Makoto Saito, Haruna Muraki, Masanobu Morioka, Takanori Teshima, Takeshi Kondo

**Affiliations:** ^1^ Blood Disorders Center Aiiku Hospital Sapporo Japan; ^2^ Department of Hematology Hokkaido University Faculty of Medicine Sapporo Japan; ^3^ Division of Laboratory Aiiku Hospital Sapporo Japan; ^4^ Sapporo Clinical Laboratory Inc. Sapporo Japan

**Keywords:** acute myeloid leukemia, booster vaccine effect, COVID‐19, myelodysplastic syndrome, SARS‐CoV‐2, vaccine

## Abstract

**Background:**

We have reported that seroconversion rates after the second dose of mRNA‐based COVID‐19 vaccines for myelodysplastic syndrome (MDS) and acute myeloid leukemia (AML) were 100% and 95% respectively, with no significant difference from healthy controls (HCs).However, there are very limited data for the response to a third vaccine dose in those patients.

**Aims:**

In this complementary study, we investigated the booster effect of a third mRNA‐based COVID‐19 vaccine dose in patients with myeloid malignancies.

**Materials & Methods:**

A total 58 patients including 20 patients with MDS and 38 patients with AML were enrolled. Anti‐SARS‐CoV‐2S immunoassays were performed at 3, 6, and 9 months after the second vaccine dose.

**Results:**

Seventy‐five percent of the MDS patients and 37% of the AML patients were receiving active treatment at the time of the third vaccination. Both the initial and third vaccine response in AML patients were comparable to those in HCs. In MDS patients, although the initial vaccine immunogenicity was inferior to that in HCs and AML patients, the third vaccine improved the response to a level not inferior to those in HCs and AML patients. Of note, the third vaccine resulted in a significant increase of antibodies in actively treated MDS patients who had shown a response inferior to that in untreated patients after two doses of vaccination.

**Discussion:**

In patients with myeloid malignancies, the third vaccine dose showed a booster effect, and disease‐ and therapy‐related factors associated with the booster response have been identified.

**Conclusion:**

The third dose of an mRNA‐based COVID‐19 vaccine showed a booster effect in patients with myeloid malignancies. Such a good booster response has not been reported in other haematological malignancies.

## Graphical abstract

The third dose of an mRNA‐based COVID‐19 vaccine showed a booster effect in patients with myeloid malignancies. The third vaccine resulted in a significantly improved response even in actively treated MDS patients who had shown an inferior response.

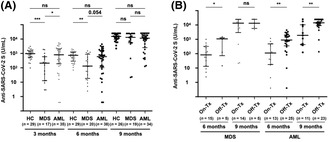

## 1 INTRODUCTION

The coronavirus disease‐2019 (COVID‐19) global pandemic caused by severe acute respiratory syndrome coronavirus 2 (SARS‐CoV‐2) is still ongoing. The development of mRNA‐based COVID‐19 vaccines has been an effective public health measure for reducing the risk of infection and severe complications from COVID‐19.^1^, ^2^ However, as patients with hematological malignancies were excluded from pivotal trials,^1^, ^2^, ^3^, ^4^, ^5^ COVID‐19 vaccine response data for those patients have been reported only recently.^6^, ^7^, ^8^, ^9^

We have reported that patients with myeloid malignancies were more responsive to the second dose of an mRNA‐based COVID‐19 vaccine than patients with lymphoid malignancies.^10^ However, there are very limited data for the response to a third vaccine dose in those patients. We therefore investigated the antibody titers against SARS‐CoV2 in patients with myeloid malignancies who received the third mRNA‐based COVID‐19 vaccine dose and compared them to those in healthy controls (HCs).

## 2 PATIENTS AND METHODS

### 2.1 Patients and healthy controls

Patients with previously treated, actively treated at the time of vaccination, and newly diagnosed myelodysplastic syndrome (MDS) or acute myeloid leukemia (AML) were included in this study. All of the patients were vaccinated with at least 2 doses of an mRNA‐based COVID‐19 vaccine, either BNT162b2 (Pfizer/BioNTech) or mRNA‐1273 (Moderna), and visited the Blood Disorders Center at Aiiku Hospital during the period from 17 August 2021 to 15 July 2022. The response criteria in patients with MDS and patients with AML were defined according to the modified International Working Group 2006 response criteria for MDS^11^ and European LeukemiaNet recommendations,^12^ respectively. All disease statuses were determined at the time of the second vaccination. We recruited healthcare workers aged 50 years or older who had received at least 2 doses of BNT162b2 vaccine as HCs. They had minimal risk of SARS‐CoV‐2 transmission from inpatients as our hospital did not accept COVID‐19 patients. Individuals with a known history of COVID‐19 were excluded from both cohorts of patients and HCs.

### 2.2 Assessment of serological response

Anti‐SARS‐CoV‐2S immunoassays (Roche Diagnostics) were performed at 3 months ± 2 weeks, 6 months ± 4 weeks, and 9 months ± 4 weeks after the second vaccine dose.^10^, ^13^ This assay has a minimum measurement value of 0.4 U/mL, with a concentration of 0.8 U/mL or more considered as a positive result, and a maximum measurement value of 25,000 U/mL.^14^ Therefore, seroconversion rate was defined as the ratio of seropositive individuals among the subjects. For individuals with antibody titer less than 0.4 U/mL or more than 25,000 U/mL, it was calculated as 0.4 U/mL or 25,000 U/mL for convenience. First and second doses of BNT162b2 and mRNA‐1273 were administered 21 and 28 days apart, respectively, with no patients being cross‐vaccinated. In Japan, the third dose was administered at least 6 months after the second dose and the choice of cross‐vaccination was made available. All third doses were administered between 6‐month and 9‐month blood samplings. Individuals who did not receive a third dose prior to the 9‐month blood sampling were excluded from the 9‐month booster effect analysis. This study was part of a prospective observation study (UMIN000045267, 000048764) and was conducted in compliance with ethical principles based on the Helsinki Declaration. This study was approved by the institutional review board of Aiiku Hospital. Written informed consent was obtained from all individuals included in the study.

### 2.3 Statistical analysis

Median antibody titers in unpaired samples of multiple groups were compared using the Mann–Whitney *U* test each, and presented in a single graph for ease of viewing. Median antibody titers in paired were compared using the Wilcoxon signed‐rank test. Antibody titers were logarithmically transformed, and geometric mean titers (GMT) with standard deviations (SD) were also calculated. The differences between two groups were evaluated by *t* test. Differences between two or more groups of categorical data were analyzed using Fisher's exact test. Two‐sided *p* < 0.05 was considered to indicate statistical significance. All statistical analyses were performed with EZR (Jichi Medical University, Saitama, Japan).^15^

## 3 RESULTS

### 3.1 Characteristics of patients and healthy controls

A total 58 patients with myeloid malignancies including 20 patients with MDS and 38 patients with AML were enrolled in this study (Table 1). HCs included 29 individuals with a median age of 55 (range: 50–72) years. There was no significant differences of gender ratio between patients with myeloid malignancies and HCs (50% females vs. 62% females, *p* = 0.47). However, median age was significantly higher for patients than for HCs (71 [interquartile range (IQR): 62–77] years vs. 55 [51–58] years, *p* < 0.000001). Furthermore, the disease‐specific analysis revealed that median age was higher than HCs (55 [51–58] years) for both MDS (vs. 75 [69–78] years, *p* < 0.0000001) and AML (vs. 69 [57–75] years, *p* < 0.001). In MDS patients, 15 patients were receiving active treatment, 4 patients were treatment‐naive observation, and one patient had been terminated active treatment at the time of third vaccination (Table 1). In AML patients, 14 patients were receiving active treatment and 24 patients were under treatment‐free observation in complete remission (CR) after completion of treatment at the time of third vaccination.

**TABLE 1:** Patients’ characteristics

	MDS (*n* = 20)	AML (*n* = 38)
Age median (range)	74.5 (59–87)	69.0 (18–88)
Sex male/female	11/9	18/20
Period from diagnosis to vaccination^a^, months, median (range)	51 (2–342)	48 (3–206)
Period from second dose to third dose^b^, days, median (range)	226 (183–257)	216.5 (180–295)
HSCT prior to vaccination	0	4

	First/second doses	Third dose	First/second doses	Third dose
Patients with seroconversion, *n* (%)	20/20 (100)	19/19 (100)	36/38 (94.7)	34/34 (100)
On active therapy at the time of vaccination				
Yes	15	15	13	14
Chemotherapy	0	1	1	1
Hypomethylating agent alone	6	6	8	2
Hypomethylating agent + Venetoclax	0	0	1	8
Targeted therapy	0	0	3	3
Erythropoiesis‐stimulating agents	5	6	0	0
Anabolic steroid	2	1	0	0
Cyclosporin A	2	1	0	0
No	5	5	25	24
Treatment off in CR	0	0	22	24
Terminated active treatment	0	1	0	0
Treatment‐naive	4	4	0	0
Before diagnosis	1	0	3	0
Status at the time of vaccination				
CR	5	6	33	34
Relapse/refractory	10	10	2	4
Previously untreated (treatment‐naive)	4	4	0	0
Before diagnosis	1	0	3	0
Vaccination rate, *n* (%)	20/20 (100)	19/20 (95.0)	38/38 (100)	34/38 (89.5)
Vaccine subtype				
BNT162b2	18	16	26	22
mRNA‐1273	2	3	12	12
Not yet vaccinated	0	1	0	4

^a^ Patients before diagnosis were excluded.

^b^ Patients who did not receive the third dose were excluded.

*Abbreviations*: AML, acute myeloid leukemia; CR, complete remission; HSCT, allogenic hematopoietic stem cell transplantation; MDS, myelodysplastic syndrome.

### 3.2 Factors affecting booster responses in patients with myeloid malignancies

**FIGURE 1:**
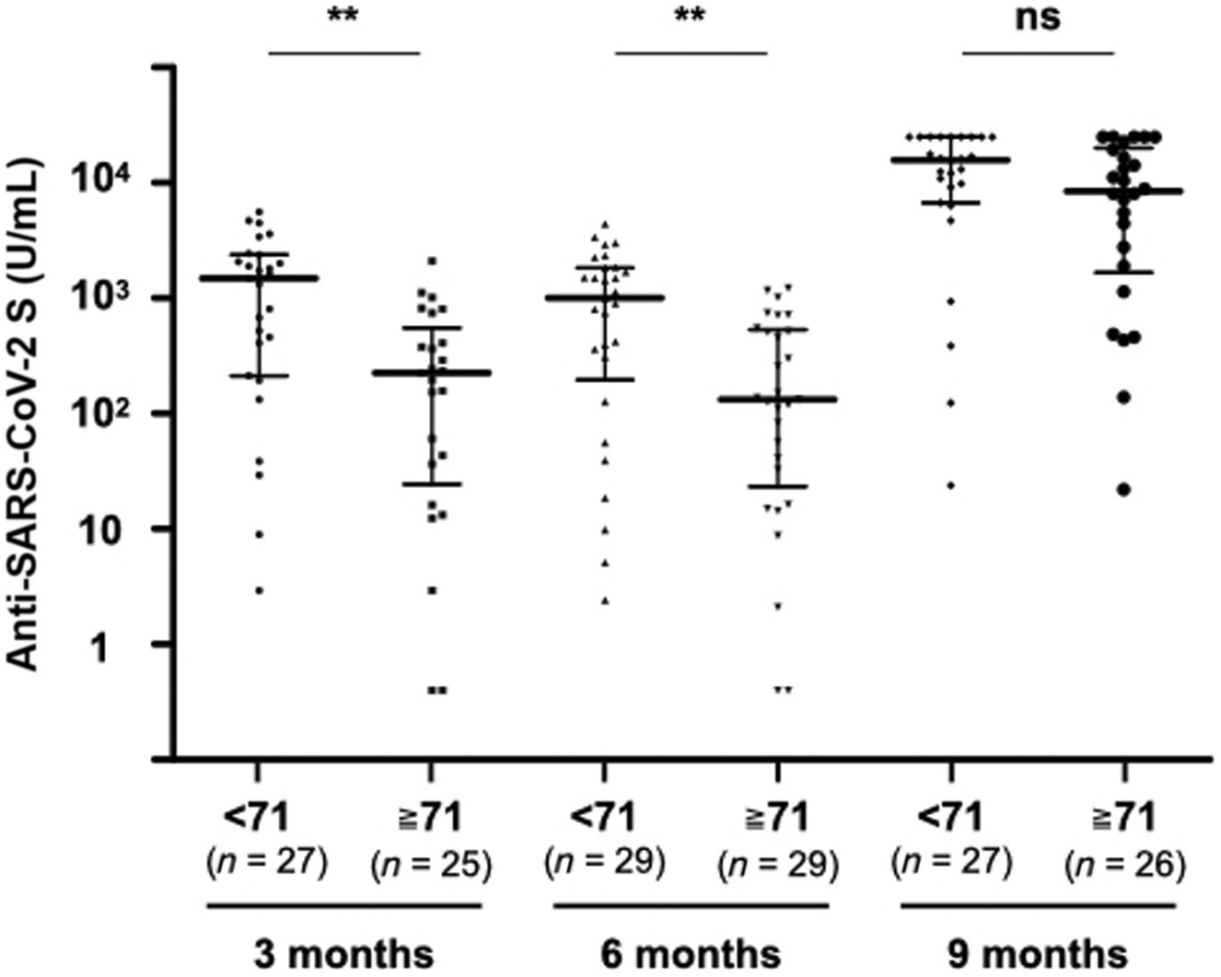
Anti‐SARS‐CoV‐2 S antibody titers after initial and third vaccination in patients with myeloid malignancies divided by median age. Patients who did not receive a third dose prior to the 9‐month blood sampling were excluded from the 9‐month booster effect analysis. The two short lines show interquartile range (IQR) and the center long line shows the median. **p < 0.01; ns, not significant.

There was no statistically significant difference in median age between MDS and AML patients (75 [69–78] years vs. 69 [57–75] years, *p* = 0.073). Therefore, we examined the association between age and antibody titers for both diseases as myeloid malignancies. The median antibody titers in patients with myeloid malignancies with a median age of 71 years or older were significantly lower than those in patients younger than 71 years at both 3 months (224 [37–411] U/mL vs. 1491 [311–2236] U/mL, *p* < 0.01) and 6 months (132 [33–517] U/mL vs. 1007 [307–1827] U/mL, *p* < 0.01) after vaccination (Figure 1). However, the median antibody titer after administration of the third vaccine in patients with myeloid malignancies with a median age of 71 years or older increased to a level comparable to that in patients younger than 71 years (8497 [2111–18,605] U/mL vs. 15,699 [7941–25,000] U/mL, *p* = 0.15). Similar result was obtained in the analysis of GMT based on log‐transformed antibody titers (Figure S1). In contrast, there were no significant differences in antibody titers divided by the median age of 55 in HCs (>55 vs. ≦55) at 3 months (945 [467–1390] U/mL vs. 1079 [666–1483] U/mL, *p* = 0.51), 6 months (643 [263–1136] U/mL vs. 748 [490–1160] U/mL, *p* = 0.62), or 9 months (19,439 [13,862–25,000] U/mL vs. 12,408 [8603–20,748] U/mL, *p* = 0.31) after the second vaccination. In patients with myeloid malignancies, there were no significant differences in antibody titers by gender after the primary vaccines or after the third vaccine.

### 3.3 Disease‐specific booster responses for MDS and AML

**FIGURE 2:**
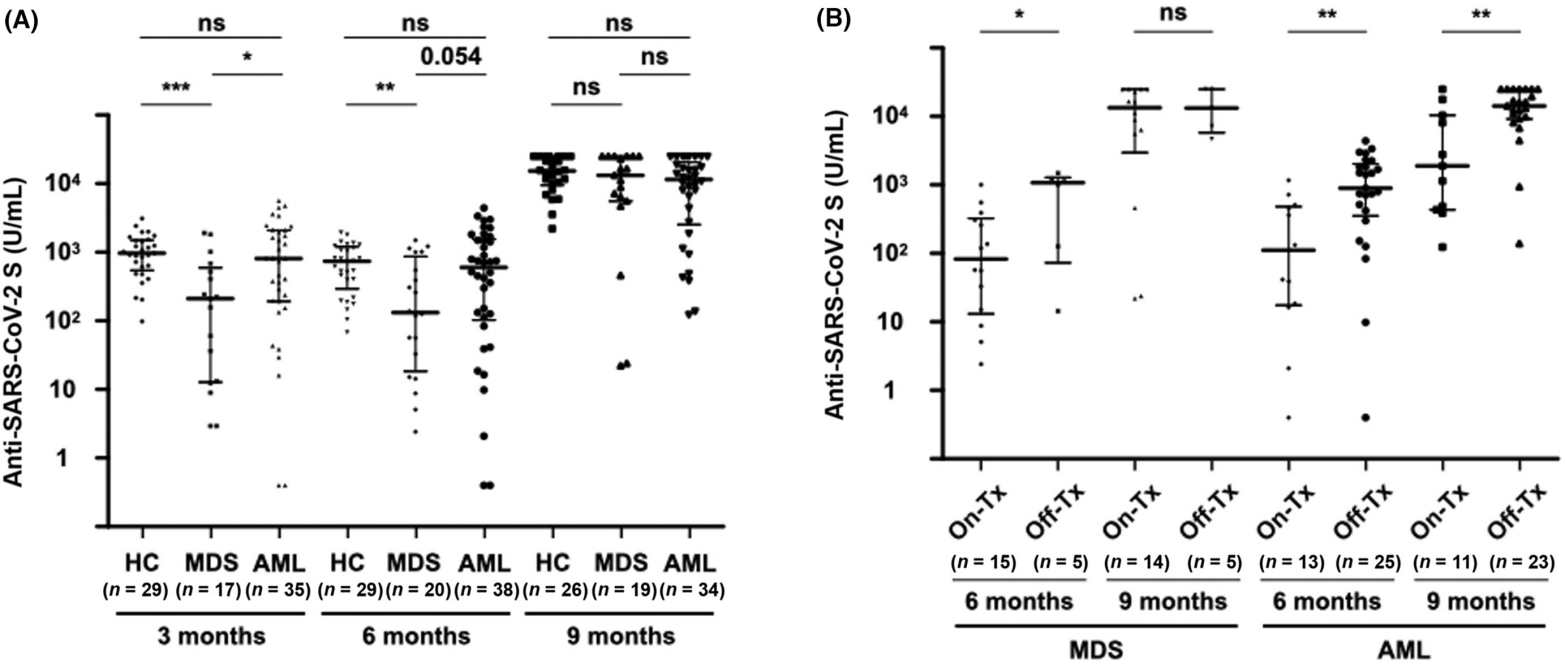
(A) Anti‐SARS‐CoV‐2 S antibody titers in healthy controls, in patients with MDS, and in patients with AML at 3, 6 and 9 months after the second vaccine dose. Individuals who did not receive a third dose prior to the 9‐month blood sampling were excluded from the 9‐month booster effect analysis. (B) Anti‐SARS‐CoV‐2 S antibody titers in patients with MDS and in patients with AML with or without treatment before and after third vaccination. In the 15 MDS patients on active treatment at 6 months after the second vaccine dose, hypomethylating agent (HMA) was administered to six patients, erythropoiesis‐stimulating agent (ESA) to five patients, anabolic steroid to two patients, and cyclosporin A to two patients. In the 14 MDS patients at 9 months, HMA was administered to five patients, ESA to 6 patients, and anabolic steroid, cyclosporin A, and low‐dose cytarabine to one patient each. In the 13 AML patients on active treatment at 6 months, consolidation chemotherapy was administered to one patient, HMA to 8 patients, HMA + venetoclax to one patient, and targeted therapy to three patients. In the 11 AML patients at 9 months, consolidation chemotherapy was administered to one patient, HMA to one patients, HMA + venetoclax to three patient, and targeted therapy to three patients. Patients who did not receive a third dose prior to the 9‐month blood sampling were excluded from the 9‐month booster effect analysis. The two short lines show interquartile range (IQR) and the center long line shows the median. *p < 0.05; **p < 0.01; ***p < 0.001; HC, healthy controls; ns, not significant; On‐Tx, on treatment; Off‐Tx, off treatment.

Seroconversion rates after the second vaccination for HCs, MDS patients, and AML patients were 100%, 100%, and 95%, respectively (*p* = 0.50). At 3 months after the second vaccination, patients with MDS showed a significantly lower antibody titer than those in HCs and AML patients (MDS 212 [13–519] U/mL, vs. HCs 974 [608–1466] U/mL, *p* < 0.001, vs. AML 806 [194–2043] U/mL, *p* < 0.05), and similar results were obtained at 6 months although the difference between the patients with MDS and AML was not significant (MDS 132 [29–666] U/mL, vs. HCs 740 [399–1163] U/mL, *p* < 0.01, vs. AML 613 [115–1499] U/mL, *p* = 0.054; Figure 2A). There was no significant difference between the antibody titers after the second vaccination in HCs and AML patients. Third vaccination rates were 90%, 95%, and 90% in HCs, MDS patients, and AML patients, respectively, and the antibody titers increased after administration of the third vaccine (Figures 2A and S2). Similar result was obtained in the analysis of GMT based on log‐transformed antibody titers (Figure S3). Of note, the antibody titer in MDS patients increased to a level not inferior to the levels in HCs and AML patients after administration of the third vaccine dose.

### 3.4 Association between treatment and booster response in patients with MDS

In MDS patients, the median antibody titer before administration of the third vaccine was significantly lower in patients on active treatment than in patients under treatment‐free or treatment‐naive observation (88 [20–295] U/mL vs. 1077 [349–1201] U/mL, *p* < 0.05; Figure 2B). However, the median antibody titer after administration of the third vaccine in MDS patients on active treatment at the time of the third vaccination increased to a level comparable to that in MDS patients under treatment‐free or treatment‐naive observation (13,703 [5749–24,465] U/mL vs. 13,284 [7166–25,000] U/mL, *p* = 0.74).

In actively treated MDS patients, the median antibody titer at 6 months was significantly lower in patients who were receiving hypomethylating agent (HMA) at the time of the initial vaccination than in patients who were receiving other treatment (12 [6–198] U/mL vs. 119 [56–552] U/mL, *p* < 0.05; Figure S4). However, after third vaccination, there was no significant difference in median antibody titers between MDS patients who were receiving HMA at the time of the third dose and those who were receiving other treatment, most of which were erythropoiesis‐stimulating agents (Table 1, Figure S4) (5550 [24–16,418] U/mL vs. 16,231 [8898–25,000] U/mL, *p* = 0.14).

### 3.5 Association between treatment and booster response in patients with AML

In contrast to MDS patients, in AML patients, not only those who were receiving treatment before the third vaccination but also those who were receiving treatment at the time of third vaccination had significantly lower antibody titers than that in patients who were under treatment‐free observation (6 months, 111 [19–454] U/mL vs. 896 [421–1853] U/mL, *p* < 0.01, 9 months, 1888 [462–9260] U/mL vs. 14,224 [9524–25,000] U/mL, *p* < 0.01; Figure 2B). Similar result was obtained in the analysis of GMT based on log‐transformed antibody titers (Figure S5).

**FIGURE 3:**
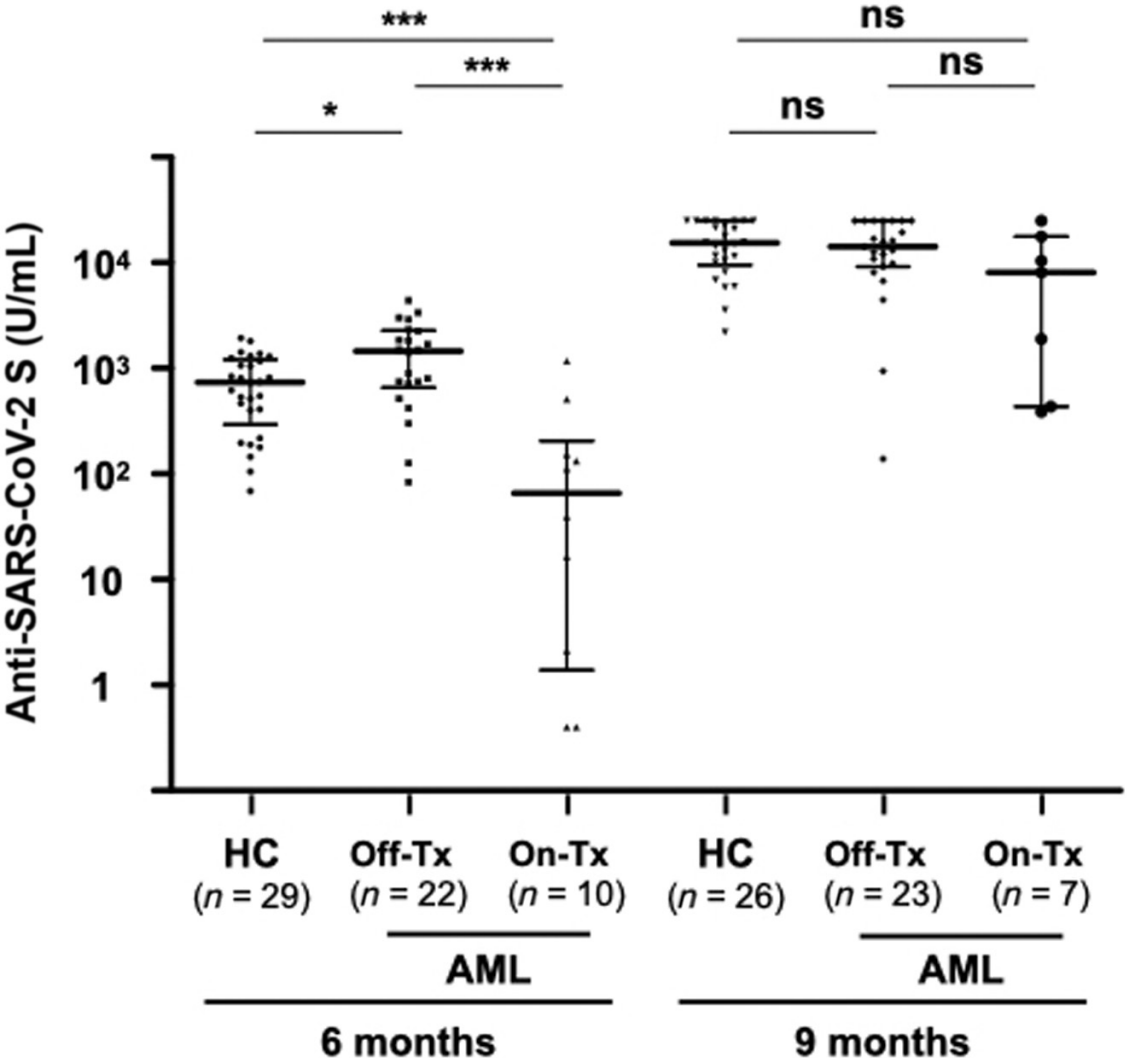
Anti‐SARS‐CoV‐2S antibody titers before and after third vaccination in healthy controls and in patients with AML in complete remission who had completed treatment or were receiving maintenance therapy at the time of initial or third vaccination. In the 10 AML patients on active treatment at 6 months, hypomethylating agent (HMA) was administered to six patients, HMA + venetoclax to one patient, and targeted therapy to three patients. In the seven AML patients at 9 months, HMA + venetoclax was administered to four patients and targeted therapy to three patients. Patients who did not receive a third dose prior to the 9‐month blood sampling were excluded from the 9‐month booster effect analysis. The two short lines show interquartile range (IQR) and the center long line shows the median. *p < 0.05; ***p < 0.001; ns, not significant; HC, healthy controls.

Among the AML patients in CR, those receiving maintenance therapy at the time of the initial vaccination had a significantly lower median antibody titer at 6 months than that in HCs or AML patients in CR after completion of treatment (75 [6–147] U/mL, vs. HCs 740 [399–1163] U/mL, *p* < 0.001, vs. patients completing treatment 1455 [718–2149] U/mL, *p* < 0.001; Figure 3). However, after the third vaccination, there was no significant difference in median antibody titers between AML patients on maintenance therapy in CR at the time of the third vaccination and HCs or AML patients who had completed treatment (8096 [1162–14,010] U/mL, vs. HCs 15,324.0 [10,122.5–24,361.3] U/mL, *p* = 0.069, vs. patients completing treatment 14,224 [9524–25,000] U/mL, *p* = 0.14). Basically, similar result was obtained in the analysis of GMT based on log‐transformed antibody titers although AML patients receiving maintenance therapy had a significantly lower GMT after third vaccination than that in HCs (Figure S6).

Similar results were obtained for HMA ± venetoclax maintenance therapy in AML patients in CR. The median antibody titer at 6 months in AML patients who were on HMA ± venetoclax maintenance therapy in CR at the time of initial vaccination was lower than the median antibody titer in HCs or AML patients who had completed treatment (39 [1–122] U/mL, vs. HCs 740 [399–1163] U/mL, *p* < 0.01, vs. patients completing treatment 1455 [718–2149] U/mL, *p* < 0.01; Figure S7). However, after the third vaccination, there was no significant difference in median antibody titers between AML patients who were in CR and receiving HMA ± venetoclax maintenance therapy at the time of the third vaccination and HCs or AML patients who had completed treatment (4266 [423–12,322] U/mL, vs. HCs 15,324 [10,123–24,361] U/mL, *p* = 0.14, vs. patients completing treatment 14,224 [9524–25,000] U/mL, *p* = 0.19). For AML patients who had completed treatment, the median antibody titer at 3 months after the second dose was comparable to that in HCs (1630 [806–2454] U/mL vs. 974 [608–1466] U/mL, *p* = 0.082), but the median antibody titer at 6 months was higher than that in HCs (1455 [718–2149] U/mL vs. 740 [399–1163] U/mL, *p* < 0.05).

## 4 DISCUSSION

Homologous and heterologous booster vaccines with BNT162b2, mRNA‐1273, and Ad26.COV2.S were shown to be immunogenic in healthy adults.^16^ In contrast, the booster vaccine immunogenicity in patients with hematological malignancies has seem to be inferior to that in healthy individuals.^17^, ^18^, ^19^, ^20^, ^21^ However, those studies focused mainly on lymphoid malignancies, in which the booster dose showed only limited increase of titer.^17^, ^18^, ^19^, ^20^ In patients with myeloid malignancies, the response of a third dose has not yet been determined and disease‐ and therapy‐related factors associated with the booster response remain unclear.

We have reported that patients with myeloid malignancies were more responsive to 2 doses of COVID‐19 vaccines than patients with lymphoid malignancies.^10^ A few later studies confirmed our results.^22^, ^23^ In this complementary study, we revealed that both the initial and third vaccine immunogenicity in AML patients, with 37% of those patients receiving active treatment, were comparable to those in HCs. In MDS patients, with 75% of those patients receiving active treatment, although the initial vaccine immunogenicity was inferior to the initial vaccine immunogenicity in HCs and AML patients, the third vaccine improved the response to a level not inferior to those in HCs and AML patients. Of note, improved third vaccine immunogenicity was observed even in MDS patients on active treatment. Such a good booster response has not been reported in other hematological malignancies.^17^, ^18^, ^19^, ^20^ However, it has been reported that vaccine immunogenicity was inferior with older age for hematological malignancies.^6^, ^9^, ^24^ In our cohort, the median age of patients with myeloid malignancies was higher than that of healthy individuals. Therefore, there is a possibility that the older age of MDS patients may contribute to their lower immunogenicity after the initial vaccination. Conversely, our results suggest that the third vaccine dose could overcome the age‐related decline in vaccine immunogenicity in MDS patients.

In contrast to MDS patients, AML patients who were being actively treated at the time of the third vaccination showed a significantly lower antibody titer after the third dose than that in AML patients under treatment‐free observation. However, a certain third vaccine response was observed in AML patients on maintenance therapy in CR. There were 13 AML patients on maintenance therapy in CR at the time of the third vaccination, and 10 of those patients were on HMA‐based therapy. Patients with MDS and patients with AML are often treated with similar HMA‐based therapy. To date, since there have been very limited data, the impact of HMA‐based therapy on COVID‐19 vaccine response is unknown.^25^ Among MDS patients, although there were six patients receiving HMA treatment, there were no patients receiving venetoclax. In contrast, among AML patients, one and eight patients received HMA + venetoclax at the time of the initial and third vaccine, respectively (Table 1). In MDS patients, vaccine response after the initial dose was reduced in patients receiving HMA therapy compared to that in patients receiving other treatments; however, the third vaccine dose overcame the effect of HMA therapy on vaccine immunogenicity. Similarly, AML patients receiving HMA‐based therapy as maintenance therapy in CR showed a reduced vaccine response after the initial dose, but this was overcome by the third vaccine dose. On the other hand, several studies have shown that venetoclax‐based treatment reduced the COVID‐19 vaccine response for patients with lymphoid malignancies.^6^, ^7^, ^17^ However, the impact of venetoclax‐based treatment on vaccine immunogenicity in patients with myeloid malignancies is still controversial.^22^, ^25^, ^26^ Because of the small number of patients in our study, it was difficult to perform any further sub‐analysis of the effect of treatment. Further studies to confirm therapy‐related factors associated with booster vaccine response are needed.

Our study has several limitations. This study included a heterogenous patient population and the number of patients was small. Probably because of these factors, the results obtained from the examination of median antibody titers and GMT were basically similar, but lost significance in some of the GMT analyses. Anti‐SARS‐CoV‐2S immunoassays has a maximum measurement value of 25,000 U/mL and antibody titer more than 25,000 U/mL was calculated as 25,000 U/mL. However, there were individuals who had antibody titers of more than 25,000 U/mL after the third vaccine dose, which may have affected the comparison of antibody titers after the third vaccination. Only humoral immunity was evaluated in this study; functional antibodies and T cell immunity were not evaluated. This study did not evaluate immune responses to SARS CoV‐2 variants. Furthermore, healthy controls are health care workers. Although our hospital had not accepted COVID‐19 patients, it is undeniable that they had asymptomatic COVID‐19 infections. In addition, we did not determine past infection status using N antibody during the course of the study. As the number of patients became even smaller in analysis by vaccine subtype, we did not evaluate responses based upon type of mRNA vaccine used. Therefore, our results should be confirmed by further prospective large‐scale studies.

In conclusion, the third dose of an mRNA‐based COVID‐19 vaccine showed a booster effect in patients with myeloid malignancies. In patients with myeloid malignancies, the initial vaccine response was inferior in older patients than in younger patients; however, the third vaccine overcame the age‐related decline in vaccine immunogenicity. Furthermore, administration of the third vaccine resulted in a significant improvement in response in MDS patients who had shown an inferior response after 2 doses of vaccination, even in MDS patients who were receiving active treatment.

## AUTHOR CONTRIBUTIONS

**Akio Mori:** Conceptualization (equal); data curation (lead); formal analysis (lead); investigation (lead); writing – original draft (lead). **Masahiro Onozawa:** Validation (lead); writing – review and editing (lead). **Mirei Kobayashi:** Data curation (equal). **Shihori Tsukamoto:** Data curation (equal). **Hajime Senjo:** Data curation (equal). **Takashi Ishio:** Data curation (equal). **Emi Yokoyama:** Data curation (equal). **Minoru Kanaya:** Data curation (equal). **Koh Izumiyama:** Data curation (equal). **Makoto Saito:** Data curation (equal). **Haruna Muraki:** Data curation (equal); investigation (equal). **Masanobu Morioka:** Data curation (equal). **Teshima Takanori:** Supervision (supporting); writing – review and editing (supporting). **Takeshi Kondo:** Conceptualization (equal); project administration (lead); supervision (lead).

## CONFLICT OF INTEREST STATEMENT

The authors declare that they have no conflict of interest.

## Supporting information


Figure S1.

Figure S2.

Figure S3.

Figure S4.

Figure S5.

Figure S6.

Figure S7.

## Data Availability

The datasets generated and/or analyzed during the current study are available from the corresponding author upon reasonable request.
