# Supplementary figures and images for: Booster effect of a third mRNA‐based COVID‐19 vaccine dose in patients with myeloid malignancies
Source: Cancer Med. 2023 Jul 6;12(16):16881–8. doi: 10.1002/cam4.6314 (PMC10501249; doi:10.1002/cam4.6314)

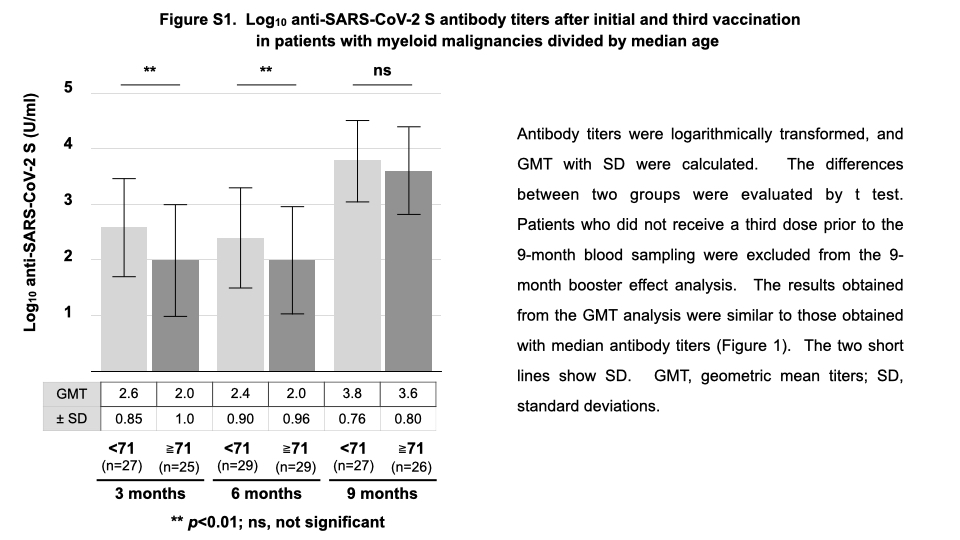

Supplement: Supplementary file 1 — Figure S1. [file CAM4-12-16881-s003.jpeg]

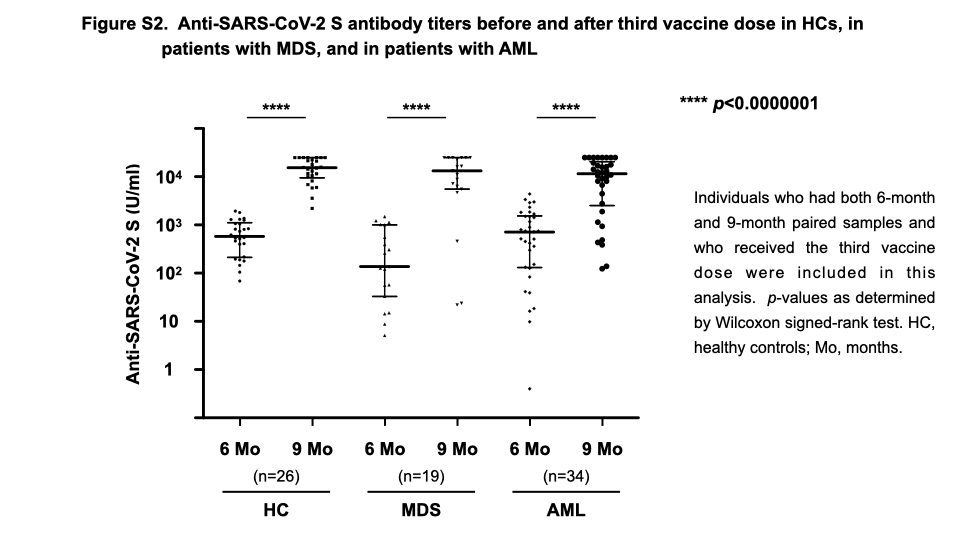

Supplement: Supplementary file 2 — Figure S2. [file CAM4-12-16881-s001.jpeg]

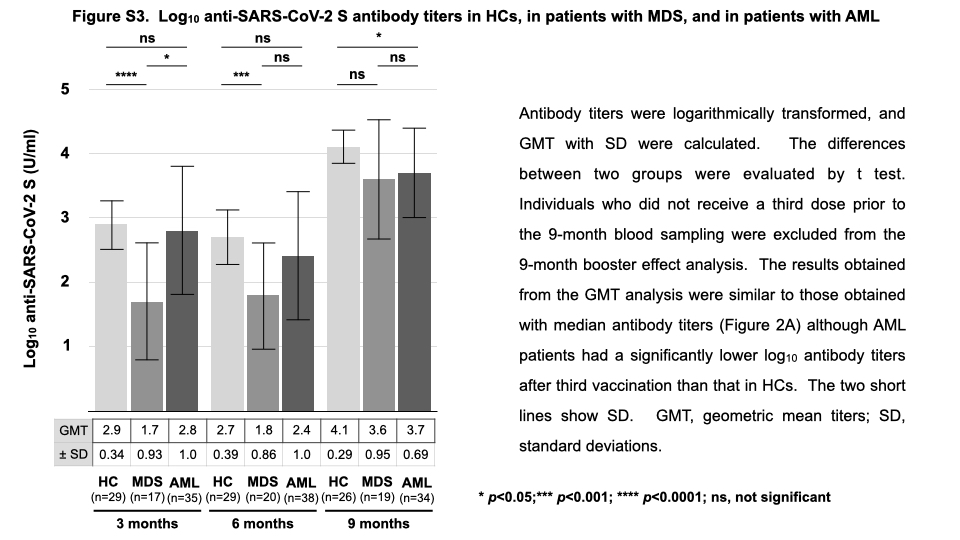

Supplement: Supplementary file 3 — Figure S3. [file CAM4-12-16881-s004.jpeg]

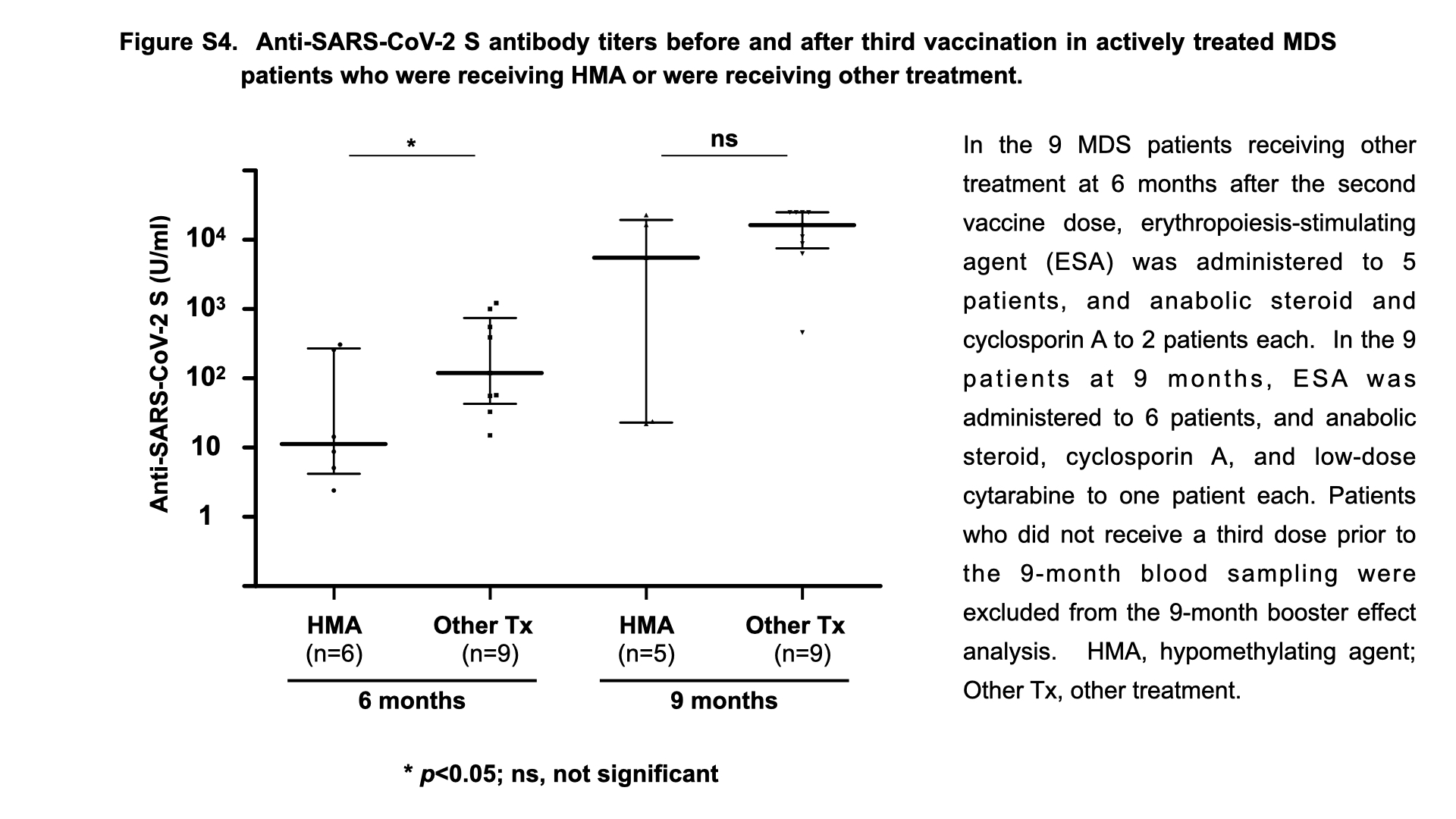

Supplement: Supplementary file 4 — Figure S4. [file CAM4-12-16881-s002.jpeg]

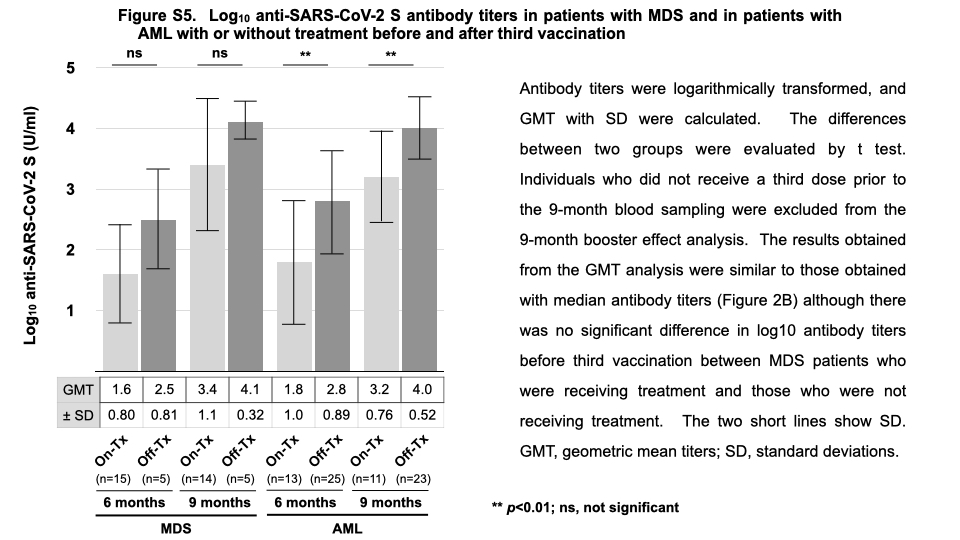

Supplement: Supplementary file 5 — Figure S5. [file CAM4-12-16881-s005.jpeg]

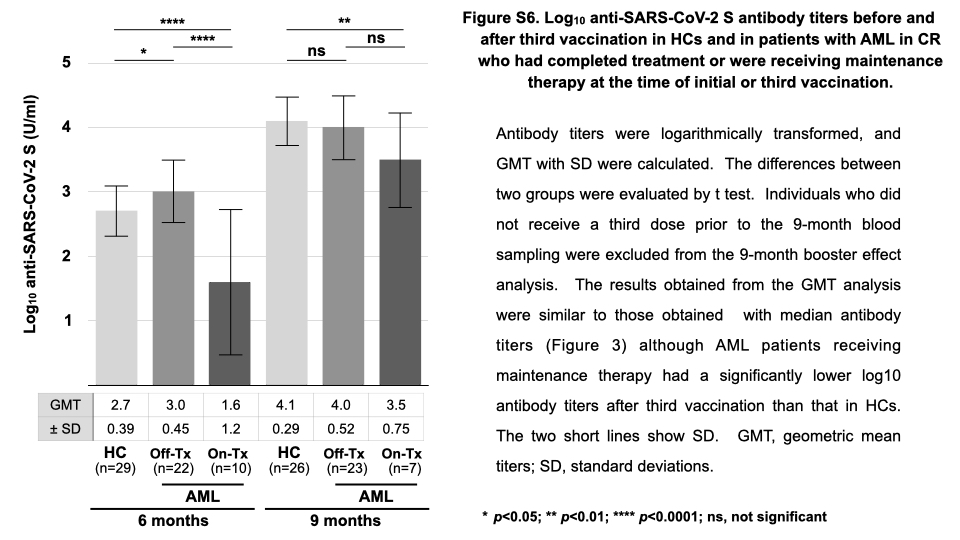

Supplement: Supplementary file 6 — Figure S6. [file CAM4-12-16881-s007.jpeg]

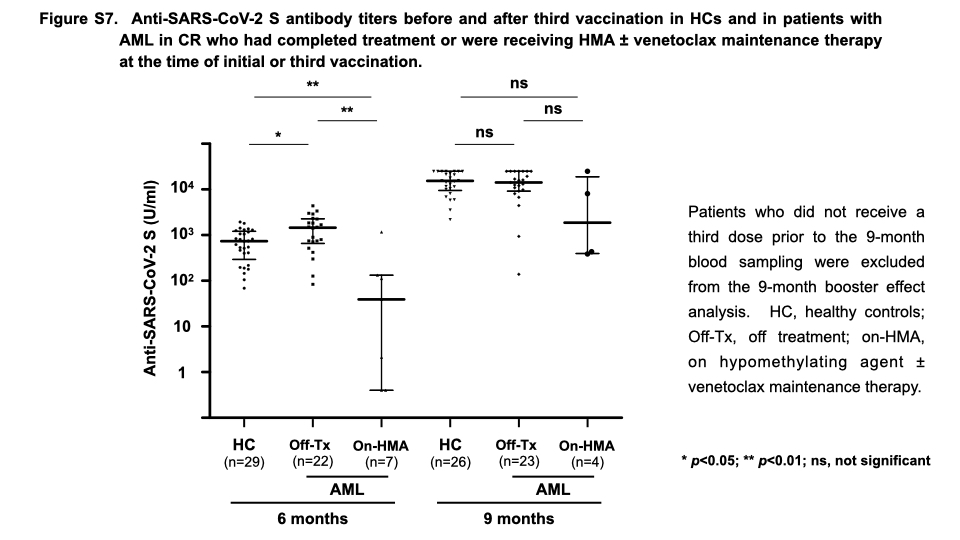

Supplement: Supplementary file 7 — Figure S7. [file CAM4-12-16881-s006.jpeg]
